# Supplementary material for: Elevating Jak-STAT signaling via SOCS3 deletion sustains photoreceptor viability and visual function in mouse models of retinitis pigmentosa
Source: Cell Commun Signal. 2026 Apr 16;24:342. doi: 10.1186/s12964-026-02878-0 (PMC13248270; doi:10.1186/s12964-026-02878-0)
Supplement: Supplementary file 1 — Supplementary Material 1: Primers for genotyping and real-time PCR. [file 12964_2026_2878_MOESM1_ESM.pdf]

Table S1. Primers for Genotyping and Real Time PCR

Experiment	Gene	Forward	Reverse
Genotyping	rd10	CTTTCTATTCTCTGTCAGCAAAGC	CATGAGTAGGGTAAACATGGTCTG
	rds (P216L)	CCTGGAGTTGCGCTGT	GTCTTTTTCATGAAGCACC
	SOCS3.fl	CGGGCAGGGGAAGAGACTGT	GGAGCCAGCGTGGATCTGC
	Rho.iCre	TCAGTGCCTGGAGTTGCGCTGTGG.	CTTAAAGGCCAGGGCCTGCTTGGC
Real Time PCR			
	Crx	GTTCAAGAATCGTAGGGCGAA	TGAGATGCCCAAAGGATCTGT
	Nr2e3	AGTCCCAGGTGATGCTAAGC	TTCTAAGATGTGCTGCCCC
	Nrl	TTCTGGTTCTGACAGTGACTACG	AAGGCTCCCGCTTTATTTC
	M-opsin	CAGTGGCAAAGCAACAGAAA	AGGGTGGGCAGTAGCAAAG
	S-opsin	TGGGATTTGTCTTCTTTGTGG	CCGAGGGATACATTGACCAG
	Rho	TCTTCTTTGGGATGGTGAGC	TGGTGGTCATTCTTCGTCCT
	Gnat1	TGACCAATAATGAAGCCATGCT	TCTTTGCCCATGGTTGATCA
	Gnat2	TCCACCTCAGTATCTGTTTTCC	TTGAGGTCAAGGAACTGACTCTT
	CLC	GACTCGTGGGGGATGTTAGC	CTAAGCTGCGGAGTTGATGCT
	CNTF	TCTGTAGCCGCTCTATCTGG	GGTACACCATCCACTGAGTCAA
	OSM	CCCTATATCCGCCTCCAAAACC	GACTCTGTCCAGTGTGGTGTAC
	Edn2	TGCGTTTTCGTCGATGCTC	GTCTGTCCCGCAGTGTTCA
	STAT3	ACCCAACAGCCGCCGTAG	CAGACTGGTTGTTTCCATTCAGAT
	SOCS3	GGAGACAGATGAGGCTGGTGA	GGACCTACTGACCGAGAGAT
	GP130	AGCATCTCCAGCAACGAGGAG	GGACAGGCTTCAGGCTGACT
	GAPDH	AGGTCGGTGTGAACGGATTTG	TGTAGACCATGTAGTTGAGGTCA
	GFAP	CCACCAAACTGGCTGATGTCTAC	TTCTCTCCAAATCCACACGAGC
