# Supplementary material for: Elevating Jak-STAT signaling via SOCS3 deletion sustains photoreceptor viability and visual function in mouse models of retinitis pigmentosa
Source: Cell Commun Signal. 2026 Apr 16;24:342. doi: 10.1186/s12964-026-02878-0 (PMC13248270; doi:10.1186/s12964-026-02878-0)
Supplement: Supplementary file 2 — Supplementary Material 2: Summary of antibodies. [file 12964_2026_2878_MOESM2_ESM.pdf]

Table S2. Summary of Antibodies

Cell Type	Antibody	Type	Dilution*	Source	Catalog Number
Primary Antibodies					
Photoreceptor	M-opsin	rAb	1:200	Chemicon	AB5405
	Peanut Agglutinin (PNA)		1:250	Vector Labs	FL-1071
	Rhodopsin (Rho4D2)	mAb	1:250	Dr. R. Molday	U. of B.C.
	Cone Arrestin	rAb	1:500	Millipore	AB15282
Signaling Molecules	pERK1/2 (Thr202/Tyr204)	rAb	1:100	Cell Signaling	9101
	pSTAT3 (Tyr705)	rAb	1:100	Cell Signaling	9171
	YAP	rAb	1:100	Cell Signaling	14074
	Cyclin D3	mAb	1:100	Cell Signaling	2936
Secondary Antibodies					
	Alexa 488 conjugated	gAb	1:500	Invitrogen	A11008
	Alexa 568 conjugated	gAb	1:500	Invitrogen	A11004

* Dilutions are for immunolabeling of sections or whole mount tissues.
